# A Consistent Procedure Using Response Surface Methodology to Identify Stiffness Properties of Connections in Machine Tools

**DOI:** 10.3390/ma11071220

**Published:** 2018-07-16

**Authors:** Jesus-Maria Hernandez-Vazquez, Iker Garitaonandia, María Helena Fernandes, Jokin Muñoa, Luis Norberto López de Lacalle

**Affiliations:** 1 Department of Mechanical Engineering, Faculty of Engineering, University of the Basque Country UPV/EHU, Plaza Ingeniero Torres Quevedo 1, E-48013 Bilbao, Spain; iker.garitaonandia@ehu.eus (I.G.); mariahelena.fernandes@ehu.eus (M.H.F); norberto.lzlacalle@ehu.eus (L.N.d.L.); 2 IK4-IDEKO, Arriaga Kalea 2, E-20870 Elgoibar, Spain; jmunoa@ideko.es

**Keywords:** stiffness properties, parameter identification, connections, machine tool, response surface methodology, design of experiments, modal testing

## Abstract

Accurate finite element models of mechanical systems are fundamental resources to perform structural analyses at the design stage. However, uncertainties in material properties, boundary conditions, or connections give rise to discrepancies between the real and predicted dynamic characteristics. Therefore, it is necessary to improve these models in order to achieve a better fit. This paper presents a systematic three-step procedure to update the finite element (FE) models of machine tools with numerous uncertainties in connections, which integrates statistical, numerical, and experimental techniques. The first step is the gradual application of fractional factorial designs, followed by an analysis of the variance to determine the significant variables that affect each dynamic response. Then, quadratic response surface meta-models, including only significant terms, which relate the design parameters to the modal responses are obtained. Finally, the values of the updated design variables are identified using the previous regression equations and experimental modal data. This work demonstrates that the integrated procedure gives rise to FE models whose dynamic responses closely agree with the experimental measurements, despite the large number of uncertainties, and at an acceptable computational cost.

## 1. Introduction

Machine tools are stationary, power-driven industrial devices used to manufacture workpieces under user and technological requirements. The most demanded requirements are accuracy and precision, which mainly depend on the static deformation and dynamic behavior of the machine tool under variable cutting forces. Assembly errors, tool trajectory errors, and the effect of thermal sources are also important issues [1]. Therefore, machine tool manufacturers devote strong efforts to perform the appropriate static, modal, and dynamic analyses of the machines, in order to determine the stresses and displacements, natural frequencies, and mode shapes. The final aim is to identify and analyze the vibration sources under different operating conditions, in order to minimize their effects on the surface finishing of the workpieces, stop the appearance of regenerative vibrations or chatter, and slow down the swift wear of the tools [2,3].

Today, the design process of modern machine tools is developed under virtual environments, where the finite element method (FEM) is widely used and particularly advised. The FEM provides a discretized model of the machine tool, whose purpose is to reproduce the real behavior of the structure. Unfortunately, this approximate model shows physical uncertainties in the material properties and loads, and numerical uncertainties in the modeling and meshing processes, limiting the quality and reliability of the results achieved by this method. In addition, the dynamic modeling of the machine tool connections is quite complicated, because of their non-linear characteristics, which are functions of the interface pressure, contact area, and surface finishes. Therefore, it is essential to devote efforts so as to improve these models, so that their dynamic characteristics resemble the real ones in the frequency range of interest.

Updating techniques [4,5] are appropriate for achieving this objective, as they allow for improving the finite element (FE) models of mechanical systems by using experimental modal data. Bais et al. [6], Houming et al. [7], and Garitaonandia et al. [8,9] have successfully applied these techniques to machine tools. Nevertheless, when the number and range of uncertainties in the FE model are large, which leads to a poor correlation with experimental data, ill-conditioning problems and non-uniqueness solutions may arise, and definitely lead to a failure in the model updating procedure. Moreover, the updating techniques are associated with high computational costs, especially those based on sensitivity calculations.

In order to solve these problems, first, it is convenient to find out which design variables have the greatest influence on the dynamic characteristics of the mechanical system. In this respect, a review of the state of the art machine tools is presented by Brecher et al. [10]. Also, in the literature [11,12,13], the most significant design variables for different types of machine tools are introduced.

An adequate technique to perform this task is the design of experiments (DoE) methodology [14] and subsequent analysis of variance (ANOVA). The DoE statistically analyzes the effect of several factors and their combinations on a process or system, and allows for determining the significant ones. Also, it is a powerful tool to bring out the interactions between the variables. 

On the other hand, an alternative option to address the time-consuming and numerical problems inherent to any iterative updating process involving FE computations, is to replace that model by an approximate model, a so-called surrogate or meta-model, which provides a more simple mathematical relationship between design variables and model responses. For instance, the coefficients of these mathematical formulations are a matter of concern for Lamikiz et al. [15], and are focused on complex new approaches for alternative processes on ruled surfaces [16].

The models developed through the response surface methodology (RSM) are widely used as meta-models [17]. This methodology is very useful in the design and optimization of new processes and products [18,19,20], especially if is affected by several variables, due to its low computational effort. Also, it can be used in an inverse sense, for the system identification applications, to find out the true values of the design variables that are inaccurately defined in a finite element model, with the help of experimental responses.

Some research related to the previous application of RSM can be found in the literature. Guo and Zhang [21] introduced the general procedure and applied it so as to update the stiffness values of three elements of the FE model of an H-shaped structure. In comparison with the traditional sensitivity-based model updating methods, the RSM-based method was found to be much more cost-efficient, providing, at the same time, accurate results. Later, Rutherford et al. [22] used RSM to perform stiffness and damping identification in two simple five degree of freedom systems, one linear and the other nonlinear. The final purpose was to demonstrate the suitability of this methodology for damage identification in civil structures. In conclusion, the procedure worked efficiently to determine the stiffness and damping coefficients in the simple linear system, while limited success was achieved in the nonlinear case.

Ren and Chen [23] updated the elastic modulus of the FE model of a full-size precast continuous box girder bridge and the cross-sectional area of two connections elements using response surface methodology. The results showed that the frequencies of the updated model were closer to the experimental ones, but there were still differences (up to 12%). Afterwards, Fang and Perera [24] used RSM to identify the structural damages in civil engineering structures. The procedure was tested on two real civil structures, a reinforced concrete frame and the I-40 bridge, and, in conclusion, it was found that the damage predictions in both structures agreed well with the experimental observations.

Recently, Sun and Cheng [25] updated the shear moduli of a honeycomb sandwich plate. That work focused on the analysis of the optimum number and position of the DoE samples, which, in conjunction with an adequate approximation algorithm, led to building the most accurate response surface model. In the end, the updated moduli were included in the FE model and the results of the dynamic analysis of this model corresponded with the experimental ones. 

A common feature of these approaches is that few design variables are identified. In this regard, Ren and Chen [23] state that RSM is still not well tested in complex structures, such as machine tools, where there are a large number of uncertain parameters and the relationships between these parameters and responses are more intricate. Another drawback is that the number of responses is small, and when the modal frequencies are selected, it is always assumed that the identified values of the design variables leads to a preservation or, even, an improvement of the correlation features between the numerical and experimental mode shapes. This is true in the modeling processes were beam elements, spring elements, and lumped masses are primarily used, leading to simple mode shapes. However, according to Fang and Perera [24], in complex structures, the correlation between the mode shapes must be taken into consideration, because in these systems, multiple (coupled) modes or different sequences between the experimental and FE modes shapes could appear. Furthermore, Gallina et al. [26] state that when changes in the values of the design variables are introduced in a mechanical system, its modal responses may be affected by degenerative phenomena, such as, mode crossing, mode veering, and mode coalescence. Therefore, it is necessary to keep this problem in mind, because otherwise the quality of the RS model could be greatly affected and, as a result, could lead to important difficulties when comparing numerical and experimental mode shapes.

Finally, in these approaches, it is assumed that all of the selected design variables affect all of the responses. This is not necessarily true, and could cause an erroneous estimation of the response surfaces, due to the presence of redundant terms in the polynomial functions.

Therefore, the aim of this paper is to present a consistent methodology so as to identify the values of the design parameters that better reproduce the dynamic responses in the mechanical systems, with a large number of uncertainties, at a reasonable computational cost, and maintaining the correlation characteristics. Firstly, parameter screening using two-level fractional factorial designs is conducted in order to determine the design variables that specifically affect each modal response, because when there are many variables, not all of them influence all of the responses. Then, second order regression equations relating the design variables and responses are attained by means of central composite design-based RSM methodology and least squares techniques. In order to look simultaneously for the best adequacy and predictive capability of these functions, the non-significant terms are removed. For that purpose, a procedure based on statistic indicators, coefficients of determination *R*^2^, and the *t*-statistic, is performed and, as a result, the number of terms in these equations is optimized. Next, using these functions, which temporarily replace the FE model, the updated values of design variables are identified by minimizing the residuals between numerical and experimental responses. In this study, a particular application of the so-called desirability function has been used to accomplish this task. Finally, the identified values are placed in the finite element model and the new dynamic responses are determined.

The proposed methodology is applied on a machining center and the comparison of the obtained results to the experimental ones demonstrates its efficiency and efficacy to update the FE models of complex mechanical systems with numerous uncertainties.

## 2. Dynamic Characteristics of the Machining Center

### 2.1. Finite Element Model

In this section, the dynamic characteristics of the DANOBATGROUP DS630 (DANOBATGROUP, Elgoibar, Spain) high speed horizontal machining center are presented. This machine tool has three linear axes and is made up of four main modules, namely, a bed frame, column, framework, and ram, which slide over roller type linear guideways. Two servo motors, directly coupled to ball-screws supported by bearings at both ends, provide the displacement along Y- and Z-axes, while the movement in the X-axis is performed by a linear motor. The machine is joined to a concrete basement by anchor bolts and leveling elements adjust and align the bed frame. 

**Figure 1:**
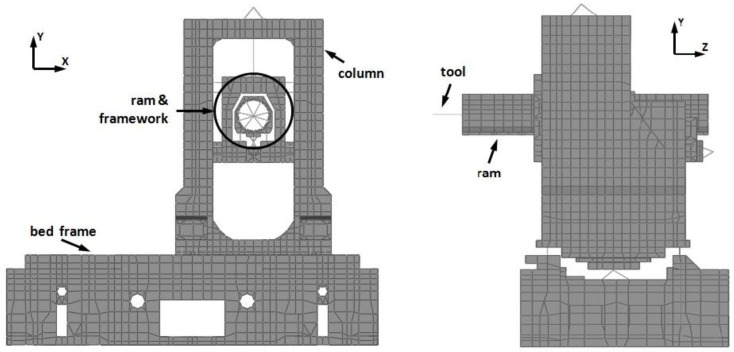
Finite element (FE) model of the machine tool.

Firstly, a FE model of the machine tool has been defined (Figure 1), which consists of 12,804 nodes and 14,983 elements, mainly shell and solid brick elements. The connections between the different components and the connection to the foundation have been modeled by linear spring elements. In this way, the contact elements and friction coefficients in the FE formulation are avoided, reducing the complexity and keeping the model linear. These linear springs characterize the previously mentioned linear guideways, ball-screws, and bolts, and are incorporated into the FE model in their locations. The anchor bolts connecting the bed frame to the basement behave rigidly, so high stiffness spring elements have been used in the modeling process. Linear guideways have been modeled assigning average stiffness values in two directions, perpendicular and transverse to the direction of movement, based on the stiffness curves provided by the guideway supplier [27], and very low stiffness values along the directions where the movement is developed. A similar modeling has been followed for the ball-screws, although, in this case, low stiffness values have been set in perpendicular and transverse directions to the main movement [2,28,29]. Moreover, the tool holder has been simulated as a beam, and spindle, servo motors and the face milling cutter as lumped masses. Finally, solid brick elements have been used to model the primary and secondary sections of the linear motor with a spring element between them. Table 1 describes the main parameter values of the FE model.

Then, using the Lanczos solver, the free motion of the structure has been analyzed by calculating the natural frequencies and mode shapes from the assembled mass and stiffness matrices of the numerical model. As the connection between the bed frame and the basement is considered in the FE model, these modal parameters correspond to the in situ configuration of the machine. According to several tests developed under chatter conditions [30], the frequency range of interest has been defined as 10 Hz to 120 Hz. The natural frequencies are shown in Table 2.

### 2.2. Experimental Modal Analysis

**Figure 2:**
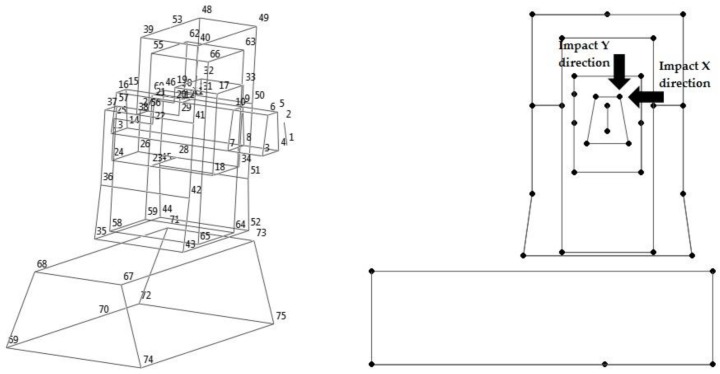
Experimental model of the machining center.

In order to experimentally determine the dynamic characteristics of the machining center, a multiple reference impact test was performed, using, as references, point 5 along X and Y directions and by exciting the system with an instrumented hammer. The translational acceleration responses in the X-, Y-, and Z-axes were measured in 75 points using triaxial accelerometers, so the accelerance frequency response functions (FRFs) corresponding to 225 degrees of freedom were obtained. The total number of measured FRFs was 450. Figure 2 shows a wire frame model representation of the test structure. The references are identified with arrows.

From the measured FRFs, a polyreference Least Squares Complex Frequency (pLSCF) estimator was used to extract the system modal parameters. Table 3 shows the natural frequencies and a brief description of the different mode shapes.

### 2.3. Comparison between FE and Experimental Modal Data

At this point, there are two sets of different results, related to the numerical and experimental models. The next step is to evaluate the correspondence between them, because it is necessary that both models show a considerable degree of correlation, in order to improve the FE model successfully.

Firstly, the geometrical correlation has been developed to match the different coordinate and unit systems used in the models, and, then, the mode shape correlation has been performed to establish a reliable pairing between the numerical and experimental modes. A very useful indicator to compare and contrast the modal vectors from the different sources is the modal assurance criterion (MAC) [31]. The MAC shows the degree of linearity between two modal vectors, *φ*_FEA_ (FEA—finite element analysis) and *φ*_exp_, as follows:

(1) $$\;MAC\left(\emptyset_{FEA},\emptyset_{exp}\right)=\frac{{\left(\emptyset_{FEA}^{T}\cdot \emptyset_{exp}\right)}^{2}}{\left(\emptyset_{FEA}^{T}\cdot \emptyset_{FEA}\right)\cdot \left(\emptyset_{exp}^{T}\cdot \emptyset_{exp}\right)}$$

The MAC can take on values from 0, showing a lack of correspondence between the modal vectors, to 1, which means that the modal vectors are the same.

Table 4 shows the frequency differences and MAC values between the FE and experimental responses, wherein the MAC values corresponding to the paired mode shapes have been bolded.

These correlation results can be considered sufficient for a large number of practical applications [32,33], as the mean frequency difference is 3.3%, and the mean MAC value is 87.0%. Nevertheless, there are still moderate differences between several natural frequencies, which confirm that it is necessary to improve the FE model. Therefore, the main goal of the following procedure will be to match the numerical frequencies to the experimental ones, while maintaining or improving the MAC pairing values.

## 3. Methods: Design of Experiments, Response Surface Methodology, and Desirability Functions

### 3.1. Two-Level Designs for Parameter Screening

Among the different types of experimental designs [14], the factorial designs are widely used to identify, at the initial stages, the main variables that affect any process or system (i.e., as screening experiments). The basic design is a two-level or 2*^k^* design, where *k* is the number of variables and each of them takes an upper and a lower level. A complete trial of such a design needs 2*^k^* runs and allows for estimating the linear effects of the *k* variables and their interactions.

Nevertheless, as the number of variables, *k*, increases, the number of runs in the trial also increases, but dramatically, and interactions between three, four, and more variables appear. Assuming that the highest interactions are negligible, it would be possible to obtain information concerning the effects of the variables and low-order interactions by running a part or fraction of the complete factorial design, 2*^k−p^*, where *p* indicates the fraction chosen (1/2*^p^*). The so-called resolution V design is especially interesting, which provides information about the contribution of variables and two-factor interactions, mixed with higher-order interactions. As these are negligible, the fractional designs are better than the complete factorial designs, because the number of runs diminishes considerably. 

Once the trial has been finished, the next step is to identify the significant factors and interactions by performing an analysis of variance on the results. According to ANOVA, the variability of the results in an experiment that is dependent on several variables, is the sum of variability due to each factor, plus that contributed by the interaction between the factors, and that added by the internal error. Also, using ANOVA, the sum of squares (SS) can be used as a measure of the overall variability, so that the greater the SS due to a factor, the larger its importance on the process or system. Thus, it will be possible to find out which variables and interactions are the most significant. 

### 3.2. Response Surface Methodology to Develop an Optimal Mathematical Model

The purpose of response surface methodology is to build an explicit function to approximate the actual relationship between the variables, *x_i_*, and a response, *y*, involved in an engineering problem. That function, preferably a low-order polynomial, is in fact a regression model, less expensive to evaluate, which can be used to predict the response developed in the system under a specific combination of variables.

In general, the behavior of the industrial processes and mechanical systems cannot be explained by linear functions [19,23,34], so, in the following section, the second-order models (Equation (2)) and the experimental designs that are preferable to adequately estimate these models will be examined.

(2) $$\;y=\beta_{0}+\overset{k}{\underset{i=1}{\sum }}\beta_{i}\cdot x_{i}+\overset{k}{\underset{i=1}{\sum }}\beta_{ii}\cdot x_{i}^{2}+\overset{k-1}{\underset{i=1}{\sum }}\overset{k}{\underset{j=i+1}{\sum }}\beta_{ij}\cdot x_{i}\cdot x_{j}+\varepsilon$$

In Equation (2), *β*_0_ is the average value of response; *y*, *β_i_*, *β_ii_*, and *β_ij_* are the partial regression coefficients; *ε* is the error term; and *k* is the number of variables.

One of the most popular designs for fitting second-order models is the central composite design (CCD). It is built in a sequential way, based on a two-level factorial (2*^k^*) design, plus 2*k* axial and *n_C_* center points. The points added to factorial design allow an efficient estimation of the possible curvature of the model. 

Firstly, a set of responses **y** is obtained on the completion the experiments of the central composite design. Then, the values of these responses and design variables are substituted in Equation (2), and rewritten in matrix form as follows:

**y** = **X** · **β** + **ε** (3)

Equation (3) is solved using the least squares method, by minimizing the sum of the squares of the errors *ε_i_*. That leads to a least squares estimator of **β**, as follows:

**b** = **(X^T^ · X)^−1^ · X^T^ · y** (4)

At this point, an initial second-order model is completely defined using all of the design variables and interactions. Then, it is necessary to perform an analysis of variance to check the significance of each parameter and the adequacy of the regression model. For this last purpose, various statistical parameters can be used, such as, the coefficient of determination *R*^2^, the adjusted *R*^2^, and the predicted *R*^2^ [17]. These coefficients are all expected to be close to 1.0, which would mean that the regression model, *y*_RSM_, explains the response, *y*, properly and that it also predicts adequately new responses. 

Nevertheless, if there are substantial differences between them, the least significant parameter is removed using the *t*-test, and a new regression model, Equation (2), is built and the analysis is repeated until the remaining parameters are all significant. On the completion of the iteration process, the optimum response surface model can be considered as adequate to carry on the next stage of the improvement procedure.

### 3.3. Identification of Updated Values of the Design Variables using the Optimum RS Model

Once the mathematical relationships between the design variables and responses have been established, the final step is to identify those values of the design variables that lead to the responses that better fit the experimental ones. This is actually an inverse multi-objective constrained optimization problem, and nonlinear programming techniques can be used to solve it.

Another alternative approach is based on the so-called desirability function [35], which is explained in the following. Firstly, each estimated response, *y*_RSM*i*_, is turned into a desirability function, *d_i_*, as follows:

(5) $$d_{i}={\left(\frac{y_{\mathrm{RSM}i}-y_{\mathrm{LOW}i}}{y_{\mathrm{OBJ}i}-y_{\mathrm{LOW}i}}\right)}^{S},\;y_{\mathrm{LOW}i}\;<\;y_{\mathrm{RSM}i}\;<\;y_{\mathrm{OBJ}i}$$

(6) $$d_{i}={\left(\frac{y_{\mathrm{RSM}i}-y_{\mathrm{UP}i}}{y_{\mathrm{OBJ}i}-y_{\mathrm{UP}i}}\right)}^{T},\;y_{\mathrm{OBJ}i}\;<\;y_{\mathrm{RSM}i}\;<\;y_{\mathrm{UP}i}$$

**Figure 3:**
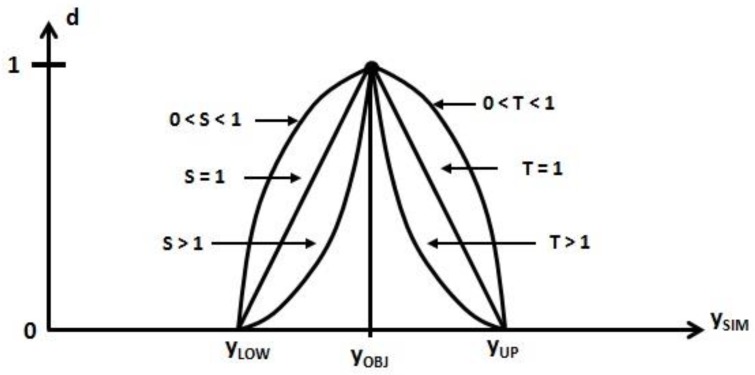
Individual desirability function.

*d_i_* = 0, *y*_RSM*i*_ < *y*_LOW*i*_ and *y*_UP*i*_ < *y*_RSM*i*_ (7)

where *y*_OBJ*i*_ is the target experimental response, and *y*_LOW*i*_ and *y*_UP*i*_ are the lower and upper limits for each response (Figure 3).

Then, a global desirability function, *D*, is built as the geometric mean of individual desirabilities, *d_i_*, as follows:

*D* = *(d_1_ · d_2_ · … · d_u_)*^1/*u*^ (8)

where *u* is the total number of the experimental responses.

Finally, the results are ranked in decreasing desirability order and the values of the design variables that maximize the global desirability *D* are selected.

## 4. Case Study

### 4.1. Initial Selection of Candidate Design Variables

**Figure 4:**
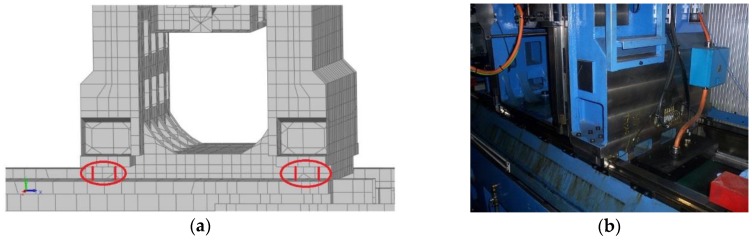
Connections between bed frame and column in a (**a**) FE model and (**b**) photograph.

**Figure 5:**
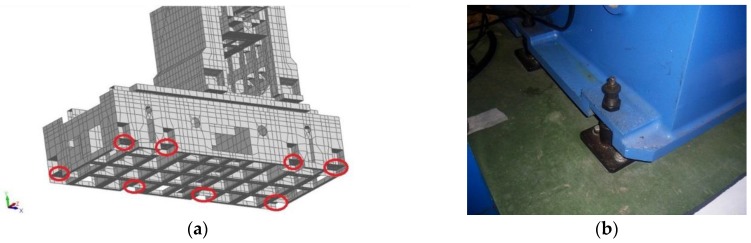
Supporting mounts of the machining center in a (**a**) FE model and (**b**) photograph.

In order to improve the FE model, first, it is necessary to select the design variables to work with. There are a large number of design parameters to be considered in this machining center, but, in fact, the main uncertainties in the FE model are concentrated on connections, as follows:

1. Stiffness values of the connection elements between main components of the machine tool (Figure 4);
2. Stiffness values assigned to the elements that attach the machine tool to the foundation (Figure 5); and
3. Stiffness value along X direction of the connection element, between the primary and secondary sections of the linear motor.

Nevertheless, the number of variables is still large, three stiffness values for the joints to the foundation, the stiffness value for the inner connection of the linear motor, and six stiffness values for the connections between the modules of the machining center (Table 5). Therefore, first, it is necessary to determine which variables affect, to a large extent, each model response and, hence, remove those whose influence is negligible. For this purpose, a resolution V design would be the most convenient (i.e., in our case, a 2^10−3^ fractional design with 128 runs) [14]. However, it is still too laborious to manage such a number of runs. Furthermore, some parameter combinations could lead to inappropriate model responses, due to the presence of the constraints among them. Thus, in order to facilitate the analysis and gain a progressive comprehension of the significance of each design variable and interaction, instead of a single 2^10−3^ fractional design, an alternative trial with seven 2^5−1^ designs (16 runs each) has been performed (Table 5). Each variable has been paired up with the rest at least once, so that after the completion of the whole set of 2^5−1^ experiments, it has been possible to look into the effects of all of the design variables and two-factor interactions by means of ANOVA.

Prior to conducting the fractional designs, the range of each variable was decided (Table 5) according to load–deformation curves [27] and previous works [30].

**Figure 6:**
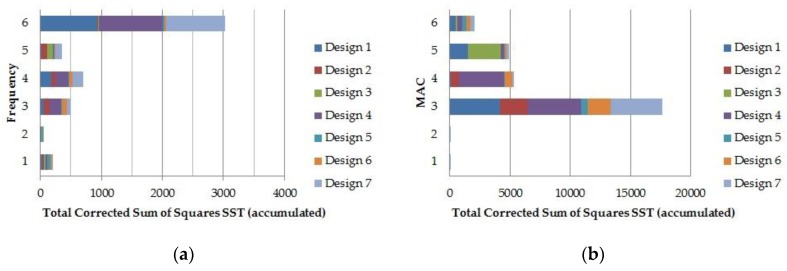
Total corrected sum of squares (SS*_T_*) for natural frequencies (**a**) and modal assurance criterion (MAC) values (**b**).

**Figure 7:**
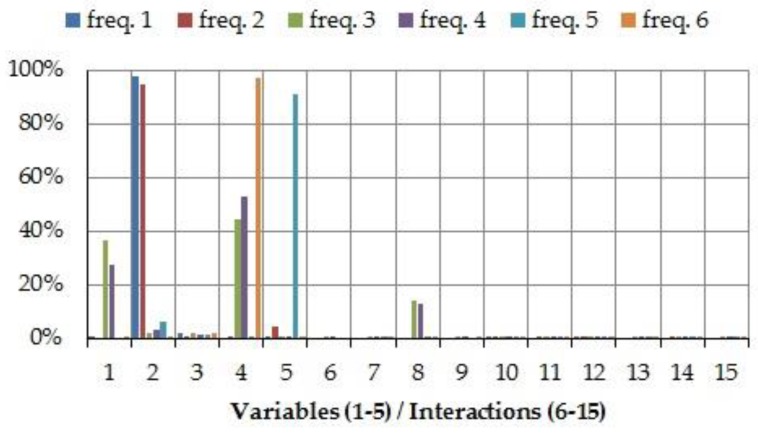
Percentage contribution for natural frequencies in design number 4: 1–5, design variables *k_X_*_210_, *k_Y_*_3_, *k_Z_*_4_, *k_X_*_8_, and *k_Y_*_9_, respectively; 6–15, two factor interactions *k_X_*_21_–*k_Y_*_3_, *k_X_*_210_–*k_Z_*_4_, *k_X_*_210_–*k_X_*_8_, *k_X_*_210_–*k_Y_*_9_, *k_Y_*_3_–*k_Z_*_4_, *k_Y_*_3_–*k_X_*_8_, *k_Y_*_3_–*k_Y_*_9_, *k_Z_*_4_–*k_X_*_8_, *k_Z_*_4_–*k_Y_*_9_, and *k_X_*_8_–*k_Y_*_9_, respectively.

**Figure 8:**
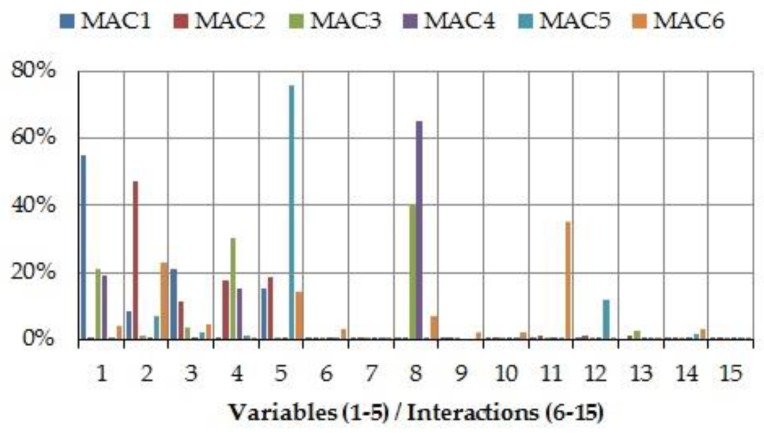
Percentage contribution for MAC values in design number 4: 1–5, design variables *k_X_*_210_, *k_Y_*_3_, *k_Z_*_4_, *k_X_*_8_, and *k_Y_*_9_, respectively; 6–15, two factor interactions *k_X_*_210_–*k_Y_*_3_, *k_X_*_210_–*k_Z_*_4_, *k_X_*_210_–*k_X_*_8_, *k_X_*_210_–*k_Y_*_9_, *k_Y_*_3_–*k_Z_*_4_, *k_Y_*_3_–*k_X_*_8_, *k_Y_*_3_–*k_Y_*_9_, *k_Z_*_4_–*k_X_*_8_, *k_Z_*_4_–*k_Y_*_9_, and *k_X_*_8_–*k_Y_*_9_, respectively.

On completion of the trial, the total corrected sum of squares, SS*_T_* (Figure 6), and the sum of squares of each factor and the two-factor interactions, mixed with higher interactions (SS*_i_* and SS*_ij_*) (Figure 7 and Figure 8), have been obtained as a measure of the variability, for all of the frequencies and MAC values. Firstly, for each response, the SS*_T_* has been examined, as some designs add much more variability than others, because of the variables involved. Thus, Figure 6 shows that *MAC*_1_ and *MAC*_2_ are not affected by the changes in the design variables, and that the variability of *f*_FEA2_ is negligible. As this frequency matches its experimental pair (Table 4), it has been omitted in later analyses.

In order to determine which variables and two-factor interactions provide the largest influence on the variability of natural frequencies and mode shapes, SS*_i_* and SS*_ij_* have been gradually analyzed in each 2^5−1^ design. As an example, Figure 7 and Figure 8 illustrate the percentage contribution of each parameter in design number 4.

From Figure 7, it is found that the design variables (1–5) have a larger influence on the natural frequencies than the two-factor interactions (6–15). For instance, design variable 2 (*k_Y_*_3_) dominates the 1st and 2nd natural frequencies, design variable 4 (*k_X_*_8_) has a huge influence on the 6th natural frequency and an important weight on the 3rd and 4th natural frequencies, and design variable 5 (*k_Y_*_9_) governs the 5th natural frequency. However, only parameter 8 (interaction *k_X_*_210_–*k_X_*_8_) slightly affects the 3rd and 4th natural frequencies. Also, design variable 3 (*k_Z_*_4_) does not seem to have a notable influence on any natural frequency.

On the other hand, from Figure 8, some of the interactions play significant roles in MAC values (3, 4, and 6, mainly). In fact, *MAC*_4_ is heavily affected by parameter 8 (interaction *k_X_*_210_–*k_X_*_8_), which also provides an important contribution to the variance of *MAC*_3_. Also, parameter 11 (interaction *k_Y_*_3_–*k_X_*_8_) causes approximately 35% of the total variability to *MAC*_6_. Nevertheless, in general, the individual design variables have a greater influence than the interactions.

This analysis has been repeated for each 2^5–1^ design, so that it has been possible to gradually gain a better insight into the influence of the design variables and two-factor interactions on the responses. Finally, those factors providing more than 99% of the total variability for the frequencies, and 97.5% for the MAC values have been selected (Table 6 and Table 7) to continue with the improvement procedure.

Several conclusions can be inferred from Table 6 and Table 7, as follows:

- The fractional factorial experiments have allowed for finding out the design variables and interactions that affect the responses. Therefore, the screening experiment has satisfactorily achieved the initial goal.
- Two design variables do not influence the natural frequencies, namely the stiffness *k_X_*_11_ and horizontal stiffness *k_X_*_21_ of the connections to the foundation.
- Three design variables are only significant for one natural frequency (*f*_FEA5_), namely the transverse stiffness *k_Z_*_63_ between the bed frame and foundations, and two stiffnesses between the modules of the machine tool, *k_Z_*_13_ and *k_Y_*_9_.
- *MAC*_5_ and *MAC*_6_ are affected by the largest number of interactions. Furthermore, some of them include design variables that do not influence them individually, for example, interaction *k_X_*_11_-*k_X_*_21_. This situation only appears in these two responses. In addition, the total number of design variables, considered both individually and in interactions, which affect each of these responses is nine (i.e., almost all). Nevertheless, along the complete set of fractional designs, the *MAC*_5_ values were always larger than 80% and the *MAC*_6_ values ranged from 68% to 73%. Thus, it has been decided not to carry on with the study of these responses, because the number of involved variables would lead to a costly analysis in the next step, while the benefits would be quite poor.
- The natural frequencies *f*_FEA3_ and *f*_FEA4_ and the corresponding MAC values depend on the same group of design variables, *k_X_*_210_, *k_Y_*_3_, *k_Z_*_4_, and *k_X_*_8_. In addition, the natural frequency *f*_FEA6_ is dependent on three of these variables, *k_Y_*_3_, *k_Z_*_4_, and *k_X_*_8_. Therefore, in the next step of the improvement process, these three natural frequencies will be analyzed together, so as to reduce the number of experiments necessary to define their meta-models. In addition, it is interesting to note that the mode shapes associated to these frequencies take place in plane XZ.
- The natural frequency *f*_FEA5_ is affected by four variables that do not have any influence on the frequencies *f*_FEA3_, *f*_FEA4_, and *f*_FEA6_, and, vice versa, the variables that affect these three frequencies do not provide any variability to the natural frequency *f*_FEA5_. Moreover, some of the design variables representing stiffness in the X direction, *k_X_*_8_ and *k_X_*_210_, do not affect the 1st and 5th mode shapes, whose principal movement is in plane YZ. Thus, it is concluded that the design variables are working collectively.

### 4.2. Development of Explicit Relationships between Design Variables and Responses

The next step of the improvement procedure is the definition of the mathematical functions that relate the variables and responses of Table 6 and Table 7, using response surface methodology. 

Taking into consideration the conclusions drawn in the previous section, referring to the collective influence of the design variables on te responses, three different central composite designs have been developed, as follows:

- Central composite (CC) design 1: including *f*_FEA1_ and design variables *k_Y_*_22_, *k_Y_*_3_, and *k_Z_*_4_. Although it would seem unnecessary to search for this relationship, because *f*_FEA1_ is already matched, as it is influenced by the design variables that also influence other frequencies, any change on them would affect this frequency too. So, it is indispensable to know this relationship.
- CC design 2: with the following responses *f*_FEA3_, *f*_FEA4_, *f*_FEA6_, *MAC*_3_, and *MAC*_4_, and design variables *k_X_*_210_, *k_Y_*_3_, *k_Z_*_4_, and *k_X_*_8_.
- CC design 3: including *f*_FEA5_ and design variables *k_Y_*_22_, *k_Z_*_63_, *k_Z_*_13_, and *k_Y_*_9_.

Each central composite design has been developed through 2*^k^* points from the factorial design with *k* factors; 2*k* axial points face centered, where one variable takes the upper and lower limits and the others have mean values; and finally one central point. Thus, a total number of 65 experiments (15, 25, and 25, respectively) have been completed. Also, prior conducting the experiments, the design variables must be normalized to values (−1), (0), and (+1), which stand for the lower bound, mean value, and upper bound of each variable, respectively (Equation (9)).

(9) $$X_{i}=2\cdot \left(\frac{k_{i}-k_{LOWi}}{k_{UPi}-k_{LOWi}}\right)-1\;$$

where *k*_UP*i*_ and *k*_LOW*i*_ are the upper and lower limits defined in Table 5.

Firstly, the study has focused on the relationship between *f*_FEA1_ and the variables that affect it. Using the results obtained from the central composite design, an initial second-order model with all of the design variables and interactions have been developed, namely Equation (10), as follows:

*f*_RSM1_ = 34.9265 + 1.1893 · *X_Y_*_3_ + 1.3810 · *X_Y_*_22_ + 0.1806 · *X_Z_*_4_ + 0.1531 · *X_Y_*_3_ · *X_Y_*_22_ + + 0.0065 · *X_Y_*_3_ · *X_Z_*_4_ + 0.0177 · *X_Y_*_22_ · *X_Z_*_4_ − 0.4593 · (*X_Y_*_3_)^2^ − 0.5171 · (*X_Y_*_22_)^2^ − 0.0559 · (*X_Z_*_4_)^2^ (10)

where *f*_RSM1_ is the estimated response corresponding to the first natural frequency, *f*_FEA1_.

Then, the significance and the predictive capability of the regression model as well as the significance of the individual regression coefficients have been examined by means of the coefficients of determination, *R*^2^ and *t*-tests (Table 8 and Table 9). 

In Table 8, the *R*^2^ coefficients for the initial model show that the regression function explains the observed responses in the central composite design experiment quite well. Also, _pred_*R*^2^ suggests that the model will fit new responses remarkably.

In order to test the significance of the different terms of Equation (10), the *t*-statistics [17] for coefficients *b_j_* of the initial model have been calculated (Table 9). Using a 95% confidence level (*α* = 0.05), these terms must be larger than the value of the *t*-distribution *t*_0*.*025,5_ = 2.571, and it is shown that the corresponding *t*-statistics for *b*_13_, *b*_23_, and *b*_33_ are smaller. Thus, these three terms are non-significant in the regression model and can be removed. As it is convenient to eliminate one term in each step, *b*_13_ has been picked out first, as its *t*-statistic was the smallest one.

Then, the same procedure, explained in previous paragraphs, has been repeated for this new model, without the term *b*_13_. The results in Table 8 (model 2) show that both _adj_*R*^2^ and _pred_*R*^2^ have increased slightly. Therefore, as expected, removing the non-significant terms in the regression model has led to a more adequate model. Nevertheless, in the regression equation still there are non-significant terms (Table 9), as some *t*-statistics are smaller than *t*_0*.*025,6_ = 2.447. So, coefficient *b*_23_ has been removed, and a new model (model 3) has been made. In this case, _adj_*R*^2^ and _pred_*R*^2^ have reduced slightly. Although the differences are totally negligible, considering that this regression model would lead to poorer results than the previous one, the iteration process has been stopped and the preceding regression model has been selected. In Table 10, the results of analysis of variance (ANOVA) of the final model are summarized.

From Table 10, it is shown that the model is highly significant (*p* < 0.001), and confirms that it can be used to simulate the response adequately.

So, the final regression equation for the first natural frequency is as follows:

*f*_RSM1_ = 34.9265 + 1.1893 · *X_Y_*_3_ + 1.3810 · *X_Y_*_22_ + 0.1806 · *X_Z_*_4_ + 0.1531 · *X_Y_*_3_ · *X_Y_*_22_ + + 0.0177 · *X_Y_*_22_ · *X_Z_*_4_ − 0.4593 · (*X_Y_*_3_)^2^ − 0.5171 · (*X_Y_*_22_)^2^ − 0.0559 · (*X_Z_*_4_)^2^ (11)

In this case, only one *b_j_* element has been removed and the optimum model is very similar to the initial one. As it will be shown later, in some regression equations, more *b_j_* elements will be eliminated, mainly those referred to in second-order terms (see, for example, Equation (17)).

A similar procedure has been followed for *f*_FEA5_. In this case, the regression equation is as follows:

*f*_RSM5_ = 87.4450 + 0.7551 · *X_Y_*_9_ + 0.5414 · *X_Y_*_22_ + 2.3563 · *X_Z_*_13_ + 0.7345 · *X_Z_*_63_ + + 0.0618 · *X_Y_*_9_ · *X_Y_*_22_ + 0.1096 · *X_Y_*_9_ · *X_Z_*_63_ + 0.0785 · *X_Y_*_22_ · *X_Z_*_63_ + 0.0725 · *X_Z_*_13_ · *X_Z_*_63_ − − 0.2318 · (*X_Y_*_9_)^2^ − 0.1674 · (*X_Y_*_22_)^2^ − 0.8645 · (*X_Z_*_13_)^2^ − 0.2321 · (*X_Z_*_63_)^2^ (12)

The coefficients of determination, *R*^2^ = 0.9989, _adj_*R*^2^ = 0.9977, and _pred_*R*^2^ = 0.9984 (Table 11), have led again to a reliable model.

Finally, the rest of the responses, *f*_FEA3_, *f*_FEA4_, and *f*_FEA6_, along with *MAC*_3_ and *MAC*_4_, have been analyzed altogether, because the variables that affect them were the same. The final regression equations are shown in Equations (13)–(17), and the coefficients of determination in Table 11.

*f*_RSM3_ = 71.1723 + 2.3373 · *X_X_*_8_ + 2.2295 · *X_X_*_210_ + 0.4979 · *X_Y_*_3_ + 0.4722 · *X_Z_*_4_ + + 1.3206 · *X_X_*_8_ · *X_X_*_210_ + 0.2019 · *X_X_*_8_ · *X_Y_*_3_ − 0.1702 · *X_X_*_210_ · *X_Y_*_3_ + 0.1562 · *X_X_*_210_ · *X_Z_*_4_ − − 2.1869 · (*X_X_*_8_)^2^ − 0.6804 · (*X_X_*_210_)^2^ (13)

*f*_RSM4_ = 76.7443 + 2.6736 · *X_X_*_8_ + 1.7965 · *X_X_*_210_ + 0.6450 · *X_Y_*_3_ + 0.5125 · *X_Z_*_4_ − − 1.3133 · *X_X_*_8_ · *X_X_*_210_ + 0.3977 · *X_X_*_8_ · *X_Z_*_4_ −0.1550 · *X_X_*_210_ · *X_Z_*_4_ − 1.2361 · (*X_X_*_8_)^2^ (14)

*f*_RSM6_ = 118.5952 + 8.1498 · *X_X_*_8_ + 0.3207 · *X_Y_*_3_ + 1.0787 · *X_Z_*_4_ − 0.1247 · *X_X_*_8_ · *X_Y_*_3_ − − 0.2232 · *X_X_*_8_ · *X_Z_*_4_ + 0.1697 · *X_Y_*_3_ · *X_Z_*_4_ − 3.2813 · (*X_X_*_8_)^2^ − 0.2193 · (*X_Y_*_3_)^2^ − 0.3628 · (*X_Z_*_4_)^2^ (15)

*MAC*_RSM3_ = 63.4984 − 9.4610 · *X_X_*_8_ + 8.9519 · *X_X_*_210_ + 2.1027 · *X_Y_*_3_ − 3.8195 · *X_Z_*_4_ + + 10.4715 · *X_X_*_8_ · *X_X_*_210_ + 1.1365 · *X_X_*_8_ · *X_Y_*_3_ − 2.6332 · *X_X_*_8_ · *X_Z_*_4_ + 3.0198 · (*X_Z_*_4_)^2^ (16)

*MAC*_RSM4_ = 84.2937 + 5.9049 · *X_X_*_8_ − 5.2622 · *X_X_*_210_ − 1.0676 · *X_Z_*_4_ + 12.2980 · *X_X_*_8_ · *X_X_*_210_ − − 9.2492 · (*X_X_*_8_)^2^ (17)

In Table 11, the coefficients of determination for *f*_RSM1_, *f*_RSM5_, and *f*_RSM6_ are very close to 1.0, while the coefficients for *f*_RSM3_ and *f*_RSM4_ are slightly lower, although greater than 0.974, and all of them are similar or better than those attained by the authors of [22,23,24]. On the other hand, the coefficients of determination for *MAC*_RSM3_ and *MAC*_RSM4_ are lower, in some cases under 0.9. In this case, it is not possible to compare them to others, because, to the best of our knowledge, in the literature, there are no results using RSM to simulate MAC responses. Nevertheless, those values are also superior to the coefficients obtained by the authors of [22,23,24] for other responses. So, in conclusion, the approximate functions in Equations (11)–(17) were judged as good enough to accurately relate the design variables and responses, and are adequate to use in the subsequent phase of the improvement procedure.

### 4.3. Determination of Updated Values of Design Variables

Once the explicit relationships between the design variables and model responses have been determined, the next step is to identify the most adequate stiffness values for the connection elements of the FE model, so that the new model simulates accurately the experimental dynamic behavior.

However, prior to performing this step, it is interesting to have a look at Table 12, which shows the responses obtained and the combinations of variables in the CC design 2, and wherein the frequencies inside the range (*f*_exp*i*_ − 1 Hz) < *f*_FEA*i*_ < (*f*_exp*i*_ + 1 Hz), and where MAC values higher than the initial ones have been bolded.

From Table 12, several conclusions can be drawn, as follows:

- The natural frequency *f*_FEA3_ approximately matches its experimental pair and, at the same time, the corresponding MAC value is higher than the initial one, only when the design variable *k_X_*_8_ is at its lower boundary. If *k_X_*_8_ takes the central or upper values, it is not possible to adequately accomplish the pairing.
- Also, the natural frequency, *f*_FEA6_, needs lower *k_X_*_8_ values to match its experimental pair.
- However, on the other side, at lower *k_X_*_8_ values, it is not viable to adjust the natural frequency *f*_FEA4_ while maintaining accurate values of MAC. Intermediate or upper values of *k_X_*_8_ are necessary to improve *f*_FEA4_, although they give rise to MAC values slightly poorer than initially.

These facts suggest that it is not possible to develop a FE model that fits those three frequencies and that provides acceptable MAC values with a unique value of design variable *k_X_*_8_. In fact, Wu et al. [36] have also addressed a similar behavior in other machine tool with roller type linear guideways. Therefore, it will be necessary to identify one *k_X_*_8_ value to match, in combination with *k_X_*_210_, *k_Y_*_3_, and *k_Z_*_4_; natural frequencies *f*_FEA3_, *f*_FEA6_; and necessarily *MAC*_3_, as well as other *k_X_*_8_ value to match *f*_FEA4_ and *MAC*_4_, taking into account that design variable *k_X_*_8_ does not affect the rest of the responses (Table 6). 

**Figure 9:**
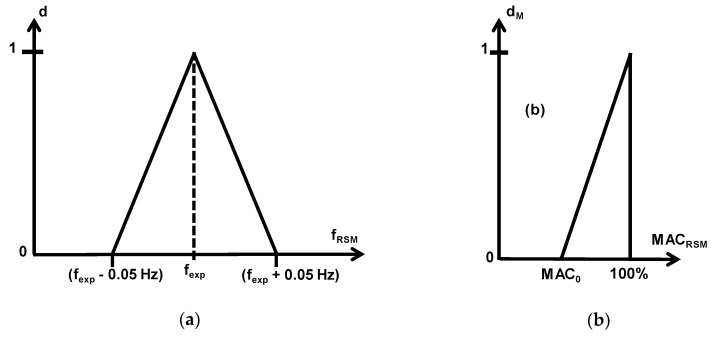
Desirability functions for natural frequencies (**a**) and MAC values (**b**).

For that purpose, the desirability function has been used, as explained in Section 3.3. Two types of desirability functions have been defined (Figure 9), one for natural frequencies and the other one for MAC values.

These functions can be expressed in mathematical form as follows:

*d_i_* = 1 − 20 · ABS(*f*_RSM*i*_ − *f*_exp*i*_), (*f*_exp*i*_ − 0.05 Hz) < *f*_RSM*i*_ < (*f*_exp*i*_ + 0.05 Hz) (18)

*d_i_* = 0, *f*_RSM*i*_ < (*f*_exp*i*_ − 0.05 Hz), *f*_RSM*i*_ > (*f*_exp*i*_ + 0.05 Hz) (19)

(20) $$d_{Mi}=1-\frac{100-MAC_{RSMi}}{100-MAC_{0}},\;MAC_{0}\;<\;MAC_{\mathrm{RSM}i}\;<\;0$$

*d_Mi_* = 0, *MAC*_RSM*i*_ < *MAC*_0_ (21)

where *MAC*_0_ has been selected taking into consideration Table 4 and Table 12.

Finally, the global desirability function *D*, Equation (22), is composed by combining one global desirability function for the frequencies, *D_f_*, and another function for the MAC values, *D_M_*, and applying weighting coefficients *w_f_* and *w_M_* to each of them.

*D* = *w_f_* · *D_f_* + *w_M_* · *D_M_* = *w_f_* · (*d*_1_ · *d*_3_ · *d*_4_ · *d*_5_ · *d*_6_)^1/5^ + *w_M_* · (*d_M_*_3_ · *d_M_*_4_)^1/2^ (22)

Table 13 shows the updated values of the normalized design variables *X_i_*, and the corresponding natural values *k_i_*. As mentioned before, two stiffness values *k_X_*_8_ have been estimated, namely: (1) is valid for the frequency ranges from 0 Hz to 72 Hz, and from 100 Hz to the upper limit of the range of interest, and (2) is adequate for the remaining range, which includes the 4th natural frequency.

These values have been driven into the FE model and the posterior FE analysis has led to the frequencies and MAC values, indicated in Table 14. For the sake of comparison, the simulated responses, *f*_RSM*i*_ and *MAC*_RSM*i*_, obtained in Equations (11)–(17), when the updated values of the design variables are substituted, are also shown.

From Table 14, it can be seen that the quadratic regression equations have provided values of the simulated frequencies that almost coincide with the values obtained after the completion of the FE analysis. In fact, the maximum distance is 0.4 Hz in the 6th frequency, which is really insignificant. The difference in the simulated MAC values is greater, which is in accordance with Table 11, where it was suggested that the predictive capability of the MAC regression equations was inferior. Therefore, both the regression meta-models and also the statistic indicators, *R*^2^ and *t*-statistic, have performed adequately.

**Figure 10:**
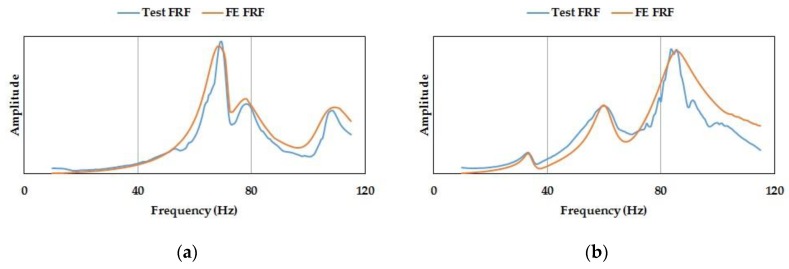
Test frequency response functions (FRFs) and synthetized FE FRF in point 5. (**a**) Response X, impact X; (**b**) response Y, impact Y.

Finally, once the identified values of design variables have been incorporated into the FE model, the resultant dynamic responses have shown a closer match to the experimental results, proving the adequacy of the conducted procedure. Thus, in Figure 10, two synthesized FRFs obtained from the updated FE model are compared to the corresponding experimental FRFs in reference point 5 (Figure 2), and the agreement is quite reasonable.

## 5. Conclusions

This paper presents a consistent methodology to improve the FE models of complex mechanical systems using, in an integrated way, different numerical and experimental techniques. The procedure is applied in a machining center with numerous uncertainties in the internal connections and supporting conditions. In this methodology, the complete design space is encompassed, so that the selection of the initial values of the design variables, which is one of the major drawbacks of the sensitivity-based methods, due to the variable sensitivity values along the design space, is avoided.

Firstly, it is demonstrated that the two-level fractional factorial design is an effective tool to perform parameter screening, as the most significant parameters and two-factor interactions are detected. For this purpose, instead of using one cumbersome resolution V design, more simple fractional designs with fewer parameters are gradually completed and examined. This procedure allows for removing high-order interactions and to circumvent the presence of constraints between the variables, and leads to a better comprehension of the influence of the design variables on the behavior of the mechanical system.

Also, in this step, it is shown that the design variables perform a kind of collective work, as groups of them affect groups of responses. In addition, some of them can be satisfactorily removed, in contrast to other findings reported in literature, where it is assumed that the complete set of the selected design variables is significant. This is a key feature of the proposed methodology, as it allows for diminishing the complexity of the subsequent regression equations, due to a substantial drop in the number of terms.

In the second step, it is demonstrated that the relationships between the stiffness parameters and the modal responses of the machine tool can be accurately expressed by second-order functions. A combined procedure using coefficients of determination and the *t*-statistic is applied to remove the non-significant terms, and thus the accuracy of regression equations is increased. At this point, the assessment of the predictive capability of the regression meta-models plays an important role. The regression equations for the MAC values are also used to ensure the correspondence between the numerical and experimental responses, because if only the frequency values are matched, it could lead to unacceptable MAC values. So far, this issue has been overlooked in the literature.

Also, the use of central composite designs allows for developing quadratic regression equations at an acceptable cost. In addition, these designs have led to detecting that the stiffness of one connection is dependent on the relative movement between the modules of the machining center.

It is proved that the quadratic regression equations are adequate to accurately identify improved values of the design variables, because when these values are included in the FE model, minimal differences between the FE and experimental responses are found. Also, because of the substitution of the full FE model by polynomial functions, the identification step, usually a costly iterative procedure, is accelerated. The use of desirability functions and weighting factors facilitates the progress of this step.

Finally, the presented methodology can be generalized to any machine tool and any design variable (damping in connections, Young’s modulus, mass density, etc.) and will allow for obtaining an updated finite element model, which would serve as a starting point to optimize the machine design and eliminate stability problems under operating conditions.

Potential future research directions include the analysis and implementation of other designs (orthogonal, Latin Hypercube, and D-optimal) for fitting second-order models with constraints in the design space. Another possible direction is the use of techniques of model reduction or transformation to modal space to diminish the time-consuming DoE runs.

## Figures and Tables

**Table 1:** Main parameter values of the finite element (FE) model.

Parameter	Value(s)	Description
Stiffness X,Y,Z	750,750,750 N/μm	Connections foundation-bed frame.
Stiffness X,Y,Z	1,720,750 N/μm	Connections bed frame-column (guideway).
Stiffness X,Y,Z	720,1,750 N/μm	Connections column-framework (guideway).
Stiffness X,Y,Z	560,750,1 N/μm	Connections framework-ram (guideway).
Stiffness X	110 N/μm	Connection between primary and secondary sections of the linear motor.
Lumped mass	120 kg	Spindle.
Lumped mass	1.5 kg	Face milling cutter.
Stiffness Y	176.7 N/μm	Y ball-screw.
Lumped mass	100 kg	Servo motor Y.
Stiffness Z	172.7 N/μm	Z ball-screw.
Lumped mass	100 kg	Servo motor Z.
*E*, *ρ*	125 GPa, 7100 kg/m^3^	Young’s modulus (*E*) and mass density (*ρ*) of the bed frame and column (cast iron).
*E*, *ρ*	175 GPa, 7100 kg/m^3^	Young’s modulus (*E*) and mass density (*ρ*) of the framework and ram (cast iron GGG70).
*E*, *ρ*	210 GPa, 7850 kg/m^3^	Young’s modulus (*E*) and mass density (*ρ*) of specific parts of the machine tool.

**Table 2:** Natural frequencies of the initial FE model. FEA—finite element analysis.

*f*_FEA1_	*f*_FEA2_	*f*_FEA3_	*f*_FEA4_	*f*_FEA5_	*f*_FEA6_
33.7	60.4	69.7	73.9	87.5	112.3

**Table 3:** Natural frequencies and mode shapes obtained by experimental modal analysis.

Mode Order	*f*_exp_ (Hz)	Damping Ratio (%)	Description of the Mode Shape
1	33.7	4.8	Rotation of the whole structure around the X-axis.
2	60.5	3.3	Translation along Y of the framework and ram.
3	65.9	3.5	Rotation of the upper part of the machine around the Y-axis.
4	77.2	5.4	Rotation of the upper part of the machine around the Y-axis, but now ram is in counter-phase.
5	84.0	5.1	Rotation of framework and ram around the X-axis.
6	106.5	3.3	Rotation of the whole structure around the Y-axis. Ram is in counter-phase.

**Table 4:** Frequency differences and modal assurance criterion (MAC) values.

FEA Order	*f*_FEA_ (Hz)	*f*_exp1_ 33.7	*f*_exp2_ 60.5	*f*_exp3_ 65.9	*f*_exp4_ 77.2	*f*_exp5_ 84.0	*f*_exp6_ 106.5	Diff. (Hz)	Diff. (%)	Pair Number
1	33.7	**96.6**	0.6	0.3	0.0	1.9	0.1	0.0	0.0	1
2	60.4	1.7	**98.8**	1.2	0.0	1.3	0.1	−0.1	−0.2	2
3	69.7	0.0	0.0	**76.3**	4.3	0.0	1.5	3.8	5.8	3
4	73.9	0.1	0.0	30.3	**89.0**	1.3	3.7	−3.3	−4.3	4
5	87.5	1.9	0.1	1.2	0.9	**91.0**	0.1	3.5	4.2	5
6	112.3	0.0	1.0	0.1	0.0	0.1	**70.3**	5.8	5.4	6

**Table 5:** List of variables used in fractional factorial designs 2^5−1^.

Connection	Design Variable	Code	Lower Bound	Nominal Value	Upper Bound	2^5–1^ Design
Foundation—bed frame	Stiffness X	*k_X_*_21_	600	750	1050	1,3,5,6
Foundation—bed frame	Stiffness Y	*k_Y_*_22_	600	750	1500	1,3,5,6
Foundation—bed frame	Stiffness Z	*k_Z_*_63_	600	750	1050	1,3,5,6
Linear motor (inner)	Stiffness X	*k_X_*_210_	80	110	160	2,4,6
Bed frame—column	Stiffness Y	*k_Y_*_3_	450	720	1125	2,4,5,7
Bed frame—column	Stiffness Z	*k_Z_*_4_	400	750	900	2,4,5,6
Column—framework	Stiffness X	*k_X_*_11_	450	720	1125	2,3,7
Column—framework	Stiffness Z	*k_Z_*_13_	400	750	900	2,3,7
Framework—ram	Stiffness X	*k_X_*_8_	210	560	900	1,4,7
Framework—ram	Stiffness Y	*k_Y_*_9_	450	750	1000	1,4,7

**Table 6:** Summary of variables affecting responses.

Connection	Design Variable	Code	*f*_FEA1_	*f*_FEA3_	*f*_FEA4_	*f*_FEA5_	*f*_FEA6_	*MAC*_3_	*MAC*_4_	*MAC*_5_	*MAC*_6_
Foundation—bed frame	Stiffness X	*k_X_*_21_									
Foundation—bed frame	Stiffness Y	*k_Y_*_22_	X			X				X	X
Foundation—bed frame	Stiffness Z	*k_Z_*_63_				X				X	X
Linear motor (inner)	Stiffness X	*k_X_*_210_		X	X			X	X		X
Bed frame—column	Stiffness Y	*k_Y_*_3_	X	X	X		X	X	X	X	X
Bed frame—column	Stiffness Z	*k_Z_*_4_	X	X	X		X	X	X		X
Column—framework	Stiffness X	*k_X_*_11_									
Column—framework	Stiffness Z	*k_Z_*_13_				X				X	X
Framework—ram	Stiffness X	*k_X_*_8_		X	X		X	X	X		X
Framework—ram	Stiffness Y	*k_Y_*_9_				X				X	X

**Table 7:** Summary of two-factor interactions affecting responses.

Responses	Interactions
*f*_FEA1_	*k_Y_*_22_–*k_Y_*_3_
*f*_FEA3_	*k_X_*_210_–*k_Y_*_3_, *k_X_*_210_–*k_Z_*_4_, *k_X_*_210_–*k_X_*_8_, *k_Y_*_3_–*k_X_*_8_
*f*_FEA4_	*k_X_*_210_–*k_Z_*_4_, *k_X_*_210_–*k_X_*_8_, *k_Z_*_4_–*k_X_*_8_
*f*_FEA5_	*k_Y_*_22_–*k_Z_*_63_, *k_Y_*_22_–*k_Y_*_9_, *k_Z_*_63_–*k_Z_*_13_, *k_Z_*_63_–*k_Y_*_9_
*f*_FEA6_	*k_Y_*_3_–*k_Z_*_4_, *k_Y_*_3_–*k_X_*_8_, *k_Z_*_4_–*k_X_*_8_
*MAC*_3_	*k_X_*_210_–*k_Y_*_3_, *k_X_*_210_–*k_Z_*_4_, *k_X_*_210_–*k_X_*_8_, *k_Y_*_3_–*k_Z_*_4_, *k_Y_*_3_–*k_X_*_8_, *k_Z_*_4_–*k_X_*_8_
*MAC*_4_	*k_X_*_210_–*k_Y_*_3_, *k_X_*_210_–*k_Z_*_4_, *k_X_*_210_–*k_X_*_8_, *k_Y_*_3_–*k_X_*_8_
*MAC*_5_	*k_X_*_21_–*k_X_*_11_, *k_X_*_21_–*k_X_*_8_, *k_Y_*_22_–*k_Z_*_63_, *k_Y_*_22_–*k_Z_*_13_, *k_Y_*_22_–*k_Y_*_9_, *k_Z_*_63_–*k_Z_*_13_, *k_Z_*_63_–*k_Y_*_9_, *k_Y_*_3_–*k_Y_*_9_, *k_Z_*_13_–*k_Y_*_9_
*MAC*_6_	*k_Y_*_22_–*k_Z_*_63_, *k_Y_*_22_–*k_Z_*_4_, *k_X_*_210_–*k_Y_*_3_, *k_X_*_210_–*k_X_*_8_, *k_Y_*_3_–*k_Z_*_13_, *k_Y_*_3_–*k_X_*_8_, *k_Z_*_4_–*k_Y_*_9_, *k_X_*_11_–*k_Y_*_9_, *k_Z_*_13_–*k_X_*_8_

**Table 8:** Significance and predictive capability of the regression models.

Coef.	Initial Model	Model 2	Model 3
*R* ^2^	0.9997	0.9997	0.9996
_adj_ *R* ^2^	0.9984	0.9986	0.9985
_pred_ *R* ^2^	0.9974	0.9982	0.9981

**Table 9:** Significance of individual regression coefficients: *t*-statistic.

Term	Coef.	Initial Model	Model 2	Model 3
Constant	*b*_0_	1410.7606	1521.1830	1482.9372
*k_Y_*_3_	*b*_1_	81.6470	88.0377	85.8242
*k_Y_*_22_	*b*_2_	94.8073	102.2280	99.6577
*k_Z_*_4_	*b*_3_	12.3961	13.3664	13.0303
*k_Y_*_3_–*k_Y_*_22_	*b*_12_	9.4043	10.1404	9.8854
*k_Y_*_3_–*k_Z_*_4_	*b*_13_	0.4007	-	-
*k_Y_*_22_–*k_Z_*_4_	*b*_23_	1.0838	1.1686	-
(*k_Y_*_3_)^2^	*b*_11_	−5.9900	−17.2415	−16.8080
(*k_Y_*_22_)^2^	*b*_22_	−18.0039	−19.4131	−18.9250
(*k_Z_*_4_)^2^	*b*_33_	−1.9461	−2.0984	−2.0457

**Table 10:** Analysis of variance (ANOVA) for the response surface model.

Source	Sum of Squares	Degree of Freedom	Mean Squares	*F* Value	*p* Value
Regression	36.114	8	4.514	2473.8	0.000
Residual	0.011	6	0.002	-	-
Total	36.125	14	2.580	-	-

**Table 11:** Summary of coefficients of determination. RSM—response surface methodology.

Coefficients of Determination	*f*_RSM1_	*f*_RSM3_	*f*_RSM4_	*f*_RSM5_	*f*_RSM6_	*MAC*_RSM3_	*MAC*_RSM4_
*R* ^2^	0.9997	0.9910	0.9870	0.9989	0.9995	0.9304	0.9271
_adj_ *R* ^2^	0.9986	0.9845	0.9805	0.9977	0.9993	0.8956	0.9080
_pred_ *R* ^2^	0.9982	0.9746	0.9750	0.9948	0.9986	0.8692	0.8816

**Table 12:** Variables and responses in central composite design 2.

Run	*f*_FEA3_	*MAC*_3_	*f*_FEA4_	*MAC*_4_	*f*_FEA6_	*k_X_*_8_	*k_X_*_210_	*k_Y_*_3_	*k_Z_*_4_
1	75.2	71.8	80.0	86.8	124.3	900	160	1125	900
2	73.8	**84.0**	78.8	88.1	121.9	900	160	1125	400
3	74.2	64.4	78.8	83.6	123.3	900	160	450	900
4	73.0	**81.3**	**77.4**	87.6	122.1	900	160	450	400
5	68.3	37.5	79.1	75.0	124.2	900	80	1125	900
6	67.9	47.6	**76.9**	78.7	121.8	900	80	1125	400
7	**66.4**	32.0	**78.2**	71.9	123.2	900	80	450	900
8	**66.1**	40.0	75.9	74.9	122.0	900	80	450	400
9	67.5	73.0	**76.8**	50.2	108.5	210	160	1125	900
10	**66.3**	72.8	**76.8**	51.3	**105.7**	210	160	1125	400
11	67.2	72.8	75.3	49.2	**107.5**	210	160	450	900
12	**66.0**	72.5	75.3	50.2	104.9	210	160	450	400
13	**66.1**	**79.1**	70.5	88.3	108.3	210	80	1125	900
14	**65.0**	**80.3**	70.3	86.4	**105.5**	210	80	1125	400
15	**65.2**	75.2	69.0	88.1	**107.3**	210	80	450	900
16	64.3	**79.8**	68.6	**89.3**	104.7	210	80	450	400
17	**66.7**	**76.4**	73.0	67.7	**107.2**	210	120	787.5	650
18	67.5	43.9	**76.3**	77.7	118.5	555	80	787.5	650
19	70.4	56.8	76.0	81.8	118.0	555	120	450	650
20	70.7	74.8	75.8	88.3	117.5	555	120	787.5	400
21	71.6	52.8	78.4	80.4	123.5	900	120	787.5	650
22	73.8	**84.1**	**78.0**	88.7	118.6	555	160	787.5	650
23	71.7	66.8	**77.1**	86.0	118.8	555	120	1125	650
24	71.5	58.3	**77.3**	82.8	119.0	555	120	787.5	900
25	71.3	63.8	**76.8**	84.7	118.5	555	120	787.5	650
Initial	69.7	76.3	73.9	89.0	112.3				
Obj	65.9	100	77.2	100	106.5				

**Table 13:** Updated values of the design variables. Units of *k_i_* are N/µm.

*X_Y_*_22_	*X_Z_*_63_	*X_X_*_210_	*X_Y_*_3_	*X_Z_*_4_	*X_Z_*_13_	*X_X_*_8_ (1)	*X_Y_*_9_	*X_X_*_8_ (2)
−0.920	−0.250	−0.695	1.000	−0.540	−0.800	−1.000	−0.300	0.485
***k_Y_*_22_**	***k_Z_*_63_**	***k_X_*_210_**	***k_Y_*_3_**	***k_Z_*_4_**	***k_Z_*_13_**	***k_X_*_8_ (1)**	***k_Y_*_9_**	***k_X_*_8_ (2)**
636	769	92	1125	515	450	210	642	722

**Table 14:** Final frequency and MAC values.

FEA Order	*f*_RSM_	*f*_FEA_	*f*_exp_	Diff. (Hz)	Diff. (%)	*MAC*	*MAC*_RSM_	*k_X_*_8_
1	33.7	33.7	33.7	0.0	0.0	96.7	-	(1)
2	-	60.5	60.5	0.0	0.0	98.7	-	(1)
3	65.9	65.8	65.9	−0.1	−0.2	78.9	76.5	(1)
4	77.2	77.0	77.2	−0.2	−0.3	80.6	83.9	(2)
5	84.0	84.1	84.0	0.1	0.1	80.9	-	(2)
6	106.5	106.1	106.5	−0.4	−0.4	70.0	-	(1)

## References

[1] Lopez de Lacalle L.N., Lamikiz A., Lopez de Lacalle L.N., Lamikiz A. (2009). Machine Tools for Removal Processes: A General View. Machine Tools for High Performance Machining.

[2] Altintas Y., Brecher C., Weck M., Witt S. (2005). Virtual machine tool. CIRP. Ann. Manuf. Technol..

[3] Quintana G., Ciurana J. (2011). Chatter in machining process: A review. Int. J. Mach. Tools Manuf..

[4] Friswell M.I., Mottershead J.E. (1995). Finite Element Model Updating in Structural Dynamics.

[5] Janter T. (1989). Construction Oriented Updating of Dynamic Finite Element Models Using Experimental Modal Data. Ph.D. Thesis.

[6] Bais R.S., Gupta A.K., Nakra B.C., Kundra T.K. (2004). Studies in dynamic design of drilling machine using updated finite element models. Mech. Mach. Theory.

[7] Houming Z., Chengyang W., Zhenyu Z. (2008). Dynamic characteristics of conjunction of lengthened shrink-fit holder and cutting tool in high-speed milling. J. Mater. Process. Technol..

[8] Garitaonandia I., Fernandes M.H., Albizuri J. (2008). Dynamic model of a centerless grinding machine based on an updated FE model. Int. J. Mach. Tools Manuf..

[9] Garitaonandia I., Fernandes M.H., Hernandez-Vazquez J.M., Ealo J.A. (2016). Prediction of dynamic behavior for different configurations in a drilling-milling machine based on substructuring analysis. J. Sound. Vib..

[10] Brecher C., Esser M., Witt S. (2009). Interaction of manufacturing process and machine tool. CIRP Ann. Manuf. Technol..

[11] Zhou Y.D., Chu L., Bi D.S. (2008). Structural optimization for hydraulic press frame. China Metal. Equip. Manuf. Technol..

[12] Li Y.B., Wu D.H., Huang M.H., Lu X.J. (2011). Design of Parallel Bearing Structure for 800 MN Forging Press with Consideration of Manufacturing Errors. Appl. Mech. Mater..

[13] Markowski T., Mucha J., Witkowski W. (2013). FEM analysis of clinching joint machine’s C-frame rigidity. Eksploat. Niezawodn. Maint. Reliab..

[14] Montgomery D.C. (2005). Design and Analysis of Experiments.

[15] Lamikiz A., Lopez de Lacalle L.N., Sanchez J.A., Bravo U. (2005). Calculation of specific cutting coefficients and geometrical aspects in sculptured surface machining. Mach. Sci. Technol..

[16] Calleja A., Bo P., Gonzalez H., Barton M., Lopez de Lacalle L.N. (2018). Highly accurate 5-axis flank CNC machining with conical tools. Int. J. Adv. Manuf. Technol..

[17] Myers R.H., Montgomery D.C., Anderson-Cook C.M. (2016). Response Surface Methodology: Process and Product Optimization Using Designed Experiments.

[18] Bonte M.H.A., van den Boogaard A.I., Huétink J. (2008). An optimization strategy for industrial metal forming processes. Struct. Multidiscip. Optim..

[19] Lü H., Yu D. (2014). Brake squeal reduction of vehicle disc brake system with interval parameters by uncertain optimization. J. Sound. Vib..

[20] Wang R., Lim P., Heng L., Mun S.D. (2017). Magnetic Abrasive Machining of Difficult-to-Cut Materials for Ultra-High-Speed Machining of AISI 304 Bars. Materials.

[21] Guo Q., Zhang L. (2004). Finite element model updating based on Response Surface Methodology. Proceedings of the 22nd International Modal Analysis Conference.

[22] Rutherford A.C., Inman D.J., Park G., Hemez F.M. (2005). Use of response surface metamodels for identification of stiffness and damping coefficients in a simple dynamic system. Shock. Vib..

[23] Ren W.-X.., Chen H.-B. (2010). Finite element model updating in structural dynamics by using the response surface method. Eng. Struct..

[24] Fang S.-E., Perera R. (2011). Damage identification by response surface based model updating using D-optimal design. Mech. Syst. Signal Process..

[25] Sun W.-Q., Cheng W. (2017). Finite element model updating of honeycomb sandwich plates using a response surface model and global optimization technique. Struct. Multidiscip. Optim..

[26] Gallina A., Pichler L., Uhl T. (2011). Enhanced meta-modelling technique for analysis of mode crossing, mode veering and mode coalescence in structural dynamics. Mech. Syst. Signal Process..

[27] Schaeffler Group. http://medias.schaeffler.de/medias/en!hp.ec.br.pr/RUE..-E-HL*RUE55-E-HL.

[28] Van Brussel H., Sas P., Németh I., de Fonseca P., van den Braembussche P. (2001). Towards a mechatronic compiler. IEEE/ASME. Trans. Mechatron..

[29] Law M., Altintas Y., Phani A.S. (2013). Rapid evaluation and optimization of machine tools with position-dependent stability. Int. J. Mach. Tools Manuf..

[30] Muñoa J. (2007). Desarrollo de un modelo general para la predicción de la estabilidad del proceso de fresado. Aplicación al fresado periférico, al planeado convencional y a la caracterización de la estabilidad dinámica de fresadoras universales. Ph.D. Thesis.

[31] Allemang R.J. (1980). Investigation of Some Multiple Input/Output Frequency Response Experimental Modal Analysis Techniques. Ph.D. Thesis.

[32] Link M., Hanke G., Maia N.M.M., Silva J.M.M. (1999). Model Quality Assesment and Model Updating. Modal Analysis and Testing.

[33] Mottershead J.E., Link M., Friswell M.I. (2011). The sensitivity method in finite element model updating: A tutorial. Mech. Syst. Signal Process..

[34] Ealo J.A., Garitaonandia I., Fernandes M.H., Hernandez-Vazquez J.M., Muñoa J. (2018). A practical study of joints in three-dimensional Inverse Receptance Coupling Substructure Analysis method in a horizontal milling machine. Int. J. Mach. Tools Manuf..

[35] Derringer G., Suich R. (1980). Simultaneous optimization of several response variables. J. Qual. Technol..

[36] Wu J., Wang J., Wang L., Li T., You Z. (2009). Study on the stiffness of a 5-dof hybrid machine tool with actuation redundancy. Mech. Mach. Theory.

